# Bilateral basal ganglia calcifications and Graves' disease in a young patient: A very rare association (case report)

**DOI:** 10.1002/ccr3.6903

**Published:** 2023-02-21

**Authors:** Lina Okar, Mhd Baraa Habib, Mohamed Khair Hamad

**Affiliations:** ^1^ Department of medical education Hamad Medical Corporation Doha Qatar; ^2^ Internal medicine department, Endocrinology division Hamad Medical Corporation Doha Qatar

**Keywords:** basal ganglia calcification, Graves' disease, hyperthyroidism, thyroid disease

## Abstract

Basal ganglia calcifications have been linked to a wide range of conditions. Mostly it is an idiopathic finding, especially in the elderly. Endocrinological and neurological disorders are two significant entities causing this radiological finding. Here, we report the first case that suggests a possible correlation between Graves' disease and basal ganglia calcifications.

## Abstract 2

Although various reasons have been correlated with the incidental finding of bilateral basal ganglia calcification, physicians should be aware of other possibilities like hyperthyroidism.

## 1 INTRODUCTION

Basal ganglia calcifications (BGCs) have been linked to various endocrinological and neurological conditions. It is also a frequent incidental finding in imaging studies.^1^ Bilateral BGCs might also reflect calcium metabolism disorders, intoxication, autoimmune and genetic diseases.^2^ It is categorized into primary and secondary brain calcifications. Among secondary causes, parathyroid disorders are the most common one. Infections are another possible cause of intracerebral calcifications.^1^

Graves' disease, an autoimmune thyroid gland disorder, that causes hyperthyroidism. It classically presents as goiter, exophthalmos, and pretibial myxedema.^3^

The occurrence of Graves' disease with bilateral BGCs has never been reported in the literature. Here, we present a young patient diagnosed with Graves' disease that causes lower limb weakness secondary to thyrotoxic periodic paralysis and found to have a computed tomography (CT) scan showing bilateral BGCs. Our case suggests a pathological correlation between Graves' disease and basal ganglia calcifications.

## 2 CASE PRESENTATION

A 27‐year‐old Filipino man who has no previously diagnosed medical illness presented to the emergency department (ED) complaining of acute bilateral leg weakness that started 2 h before the ED visit. He also had some weakness in his arms, but it was less severe. The patient denied any pain or numbness. He mentioned that he lost 5 kg over the last month.

**FIGURE 1:**
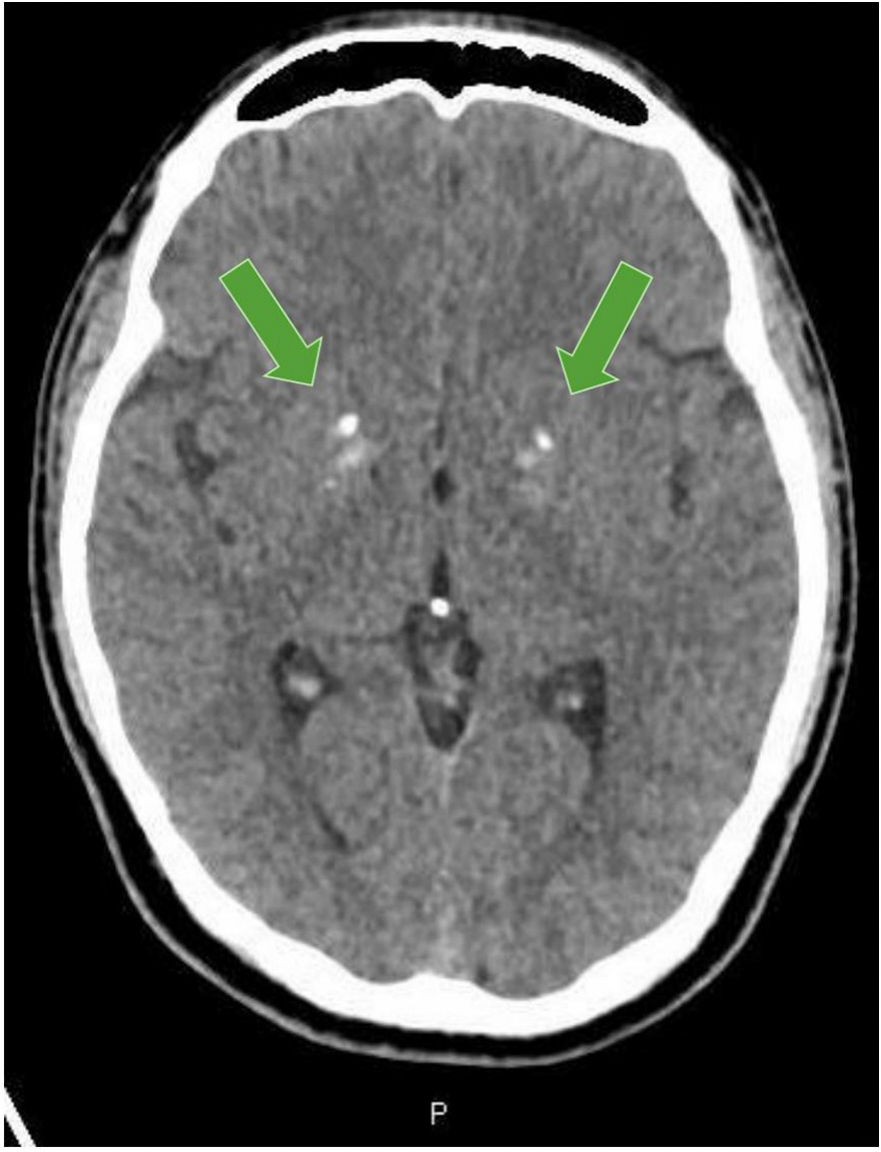
Head CT showing bilateral basal ganglia calcifications.

Vital signs were normal apart from tachycardia at 117 beats/minute. Physical examination revealed muscle power 4/5 in both upper limbs, 3/5 in both lower limbs, and diffuse goiter. ECG demonstrated sinus tachycardia. Given the acute onset of the weakness, head computed tomography was done to rule out stroke; however, it showed Bilateral basal ganglia and dentate nuclei calcifications Figure 1. Blood tests were remarkable for high FT4 > 100 mIU/L (11.0–23.3), low TSH <0.01 mIU/L (0.30–4.20), low potassium level = 3 mmol/L (3.5–5.3), and high TSH receptor antibodies (TRAP).

Based on the above findings, the patient had Graves' disease and thyrotoxic periodic paralysis. He improved dramatically after intravenous potassium replacement. He has been started on carbimazole 20 mg twice per day and propranolol 40 mg twice per day.

We raised the suspicion of a potential association between Graves' disease and basal ganglia calcification. We took a further history from the patient; he had no past remarkable central nervous system infections, no exposure to lead or other heavy metals, and no significant family history. Further workup showed normal calcium, phosphorus, and basal metabolic panel.

## 3 DISCUSSION

Basal ganglia calcification has been reported in 15%–20% of the population, mainly in patients over 65.^4^ This radiological finding presents in Fahr's disease, a rare inherited disorder, with the onset around the fourth to the fifth decade. It presents with a movement disorder, neuropsychiatric manifestations, and basal ganglia calcifications. The differential diagnosis of basal ganglia calcifications is broad and categorized into primary and secondary.^5^ Primary or idiopathic refers to the presence of radiological findings in the absence of clinical symptoms or metabolic and electrolyte panel disturbances.^6^ Secondary basal ganglia calcifications are associated with endocrinological and neurological disorders. Parathyroid disorders are the most common underlying pathology due to abnormality in calcium/phosphor balance, leading to generalized body calcifications and basal ganglia involvement. In 1980, Chad R. Cohen et al. divided secondary causes into four main categories. Endocrine causes include hypoparathyroidism, pseudohypoparathyroidism, and pseudo‐pseudohypoparathyroidism, congenital/developmental, inflammatory, and toxic. They concluded that any calcification in the basal ganglia, dentate nucleus, and multiple areas in the cortex is pathological, whatever the patient's age. At the same time, calcification in globus pallidus is considered pathological in patients under 40 years and physiological findings in those over 40 years.^7^ MG Harrington et al. included hypothyroidism among the possible causes for this radiological finding.^8^ Another article reported bilateral striocerebellar calcification associated with Hashimoto's disease. However, the mechanism is unclear; the deposits contain mucopolysaccharide colloid material which might raise suspicion of a relationship between both entities.^9^ In our case, the patient came with muscle weakness with low potassium levels. Laboratory tests revealed the presence of thyrotoxicosis with later positive TSH receptor antibodies (TRAB), which confirmed the diagnosis of Graves' disease. We did not find calcium, phosphorus, or parathyroid hormone (PTH) abnormalities. No previous reports suggest a correlation between basal ganglia calcification and Graves' disease or hyperthyroidism. We report the first case that suggests this correlation. The early age of our patient and absence of any family history of a neuropsychiatric disorder or similar findings, and the absence of any infections, travel history, or toxic exposure support the correlation in our case.

## 4 CONCLUSION

Basal ganglia calcifications are most associated with endocrinological causes. Parathyroid disorders are a well‐established cause of this radiological finding. However, thyroid disorders, especially hyperthyroidism, are not reported. We Suggest that Graves' disease might be associated with bilateral basal ganglia calcification.

## AUTHOR CONTRIBUTIONS

**Lina Adnan Okar:** Supervision; writing – original draft; writing – review and editing. **Mhd Baraa Habib:** Writing – review and editing. **Mohammad Khair Ahmad Hamad:** Writing – review and editing.

## CONFLICT OF INTEREST

The authors report no conflicts of interest.

## ETHICAL APPROVAL

Written informed consent was obtained from the patient to publish this report in accordance with the journal's patient consent policy case approved by HMC Medical Research center.

## CONSENT

Written informed consent was obtained from the patient to publish this report in accordance with the journal's patient consent policy.

## Data Availability

All data related to this case are available upon request.
